# Brain death: a clinical overview

**DOI:** 10.1186/s40560-022-00609-4

**Published:** 2022-03-16

**Authors:** William Spears, Asim Mian, David Greer

**Affiliations:** 1 Department of Neurology, Boston University, Boston Medical Center, 85 East Concord Street, Room 1145, Boston, MA 02118 USA; 2 Department of Radiology, Boston University, Boston Medical Center, 820 Harrison Avenue FGH, 3rd floor, Boston, USA

**Keywords:** Brain death, Death by neurologic criteria, Brainstem death, ECMO, Targeted temperature management, Pediatrics

## Abstract

Brain death, also commonly referred to as death by neurologic criteria, has been considered a legal definition of death for decades. Its determination involves many considerations and subtleties. In this review, we discuss the philosophy and history of brain death, its clinical determination, and special considerations. We discuss performance of the main clinical components of the brain death exam: assessment of coma, cranial nerves, motor testing, and apnea testing. We also discuss common ancillary tests, including advantages and pitfalls. Special discussion is given to extracorporeal membrane oxygenation, target temperature management, and determination of brain death in pediatric populations. Lastly, we discuss existing controversies and future directions in the field.

## History of brain death

Preceding the 1950s, the concept of death revolved around cessation of cardiorespiratory function. It naturally followed that cessation of brain function occurred after the loss of respiration and circulation, and indeed loss of brain activity was considered a critical component of death.

In the years that followed, the development of advanced live support measures including cardiopulmonary resuscitation (CPR) and positive pressure ventilation (PPV) brought this interdependence and the traditional definition of death into question. In 1959, the concept of brain death/death by neurologic criteria (BD/DNC) was first theorized as “le coma dépassé”, by Mollaret and Goulon, who described an apneic, comatose patient without brainstem reflexes or electroencephalographic (EEG) activity [1]. Neurologists began to postulate that neurologic function was equally or more vital than cardiopulmonary function, and began a process to define death neurologically, independent of other essential organ functions. In 1968, a group of Harvard faculty proposed the first clinical definition as the Harvard Brain Death Criteria, which consisted of clinical and EEG criteria [2]. In 1980, the Uniform Determination of Death Act established a legal basis for a neurologic determination of death in the U.S., and adult guidelines were put forth in the 1995 (and revised 2010) American Academy of Neurology (AAN) guidelines on the determination of BD/DNC. In 1987, the American Academy of Pediatrics task force on brain death in children published guidelines for the pediatric population [3], which was updated in 2011 [4, 5].

## What does brain death mean?

First, what does the term “brain death” truly mean? This is perhaps best understood by exploring the evolution and controversy of the idea. In fact, one of the salient remaining debates in the field involves the terminology of brain death, sometimes also referred to as “whole brain death”, or “brainstem death”. In order to promote a broad understanding by lay persons, scientists, and legal powers, most experts advocate for use of the term BD/DNC [6].

Proponents of the idea of neurologic criteria to diagnose brain death argue that the body is more than the sum of its parts, and that death is equated to loss of the whole person [7]. For example, most would not argue that the loss of a kidney, arm or leg results in death, but that a higher concept of personhood or consciousness exists, for which the brain is the principal architect [8]. Additionally, although functions of many of the body’s organ systems can be artificially supported, the brain is the main control system governing vital bodily functions including cardiorespiratory support, and that when the brain ceases to function, these vital functions will also eventually cease. In practice, diagnosis of BD/DNC is essential to organ transplantation, particularly cardiac, in that brain dead donors are the only accepted source for cardiac transplant in the United States. However, importantly, declaration of BD/DNC is an important and separate medical diagnosis that should be made independent of the need for organ transplantation.

Historic detractors of the concept of BD/DNC argued a number of points, claiming that brain death is a legal construct with the sole purpose of permitting organ donation [9], or that some individuals who have been declared brain dead can continue to grow and function in ways that are arguably inconsistent with death [10]. Some also argue that brain death cannot be declared when there is evidence of persistent neurological functioning such as small areas of the brain that appear undamaged, or persistence of neuroendocrine functioning following devastating cerebral injury.

## Definitions of death by neurologic criteria

Two of three concepts of BD/DNC exist as the dominant accepted understanding of the term. The first and most widely accepted is the “whole brain” formulation which asserts that brain death is equivalent to catastrophic injury to all the major structures of the brain including the hemispheres, diencephalon, brainstem, and cerebellum. In this view, confirmation of complete and permanent damage to the whole brain should be confirmed before BD/DNC is ultimately declared. This concept is the foundation of the original Harvard brain death criteria [2], is the formulation officially advocated by the United States (U.S.) and most other countries for which official national brain death protocols exist [11], and is formulation advocated by the World Brain Death Project, a group of leading investigators and international professional societies who aim to develop unified international recommendations and global consistency regarding the determination of BD/DNC [6]. It should be noted that this formulation does not traditionally require the loss of neuroendocrine function.

The second concept refers to “brainstem death” which is the accepted construct in the United Kingdom (U.K.) and a few other countries [11, 12], asserting that destruction of the brainstem alone is equivalent to the death of a human, given that the brainstem partially houses the centers for consciousness, as well as essential cardiac and respiratory centers. Based on this line of thinking, it logically follows that in the context of severe primary infratentorial brain injury, damage to other areas of the brain have no relevance to the diagnosis of BD/DNC.

A third but less traditional concept of brain death is the “higher brain” formulation, which postulates that only destruction of the higher brain, including the cortex and bilateral hemispheres, is necessary to diagnose BD/DNC, given these areas are critical to cognition [13]. However, patients with only loss of higher brain function maintain the ability to breathe, which is at odds with the traditional criteria for BD/DNC determination, which rely on establishment of apnea as an essential component of the clinical BD/DNC evaluation [6].

Clinically, the distinction between the “whole brain” and “brainstem” formulations of death may seem of little consequence, meaning that in the majority of devastating brain injuries from any mechanism, irreversible injury to the brainstem occurs via downward herniation following a primarily supratentorial lesion. Therefore, an injury to the whole brain is likely in most cases. Further, the traditional determination of BD/DNC will still rely on establishment of a cause of injury, exclusion of confounding conditions and reversible causes, presence of coma, loss of brainstem reflexes, and apnea [14]. Primarily infratentorial lesions such as basilar artery infarcts, primary brainstem hemorrhage, or brainstem encephalitis make up the minority of all brain death evaluations, estimated in a recent study as < 2% [15]. However, in these cases, there may be relative preservation of the cerebral hemispheres, and, although the traditional pathways of consciousness are likely disrupted to some extent, some ascending tracts may remain intact and covert consciousness may theoretically exist [16]. In these cases, conclusive statements regarding the potential for consciousness or meaningful higher order functioning should be withheld, given this has not been well studied in the setting of primary infratentorial lesions.

**Fig. 1:**
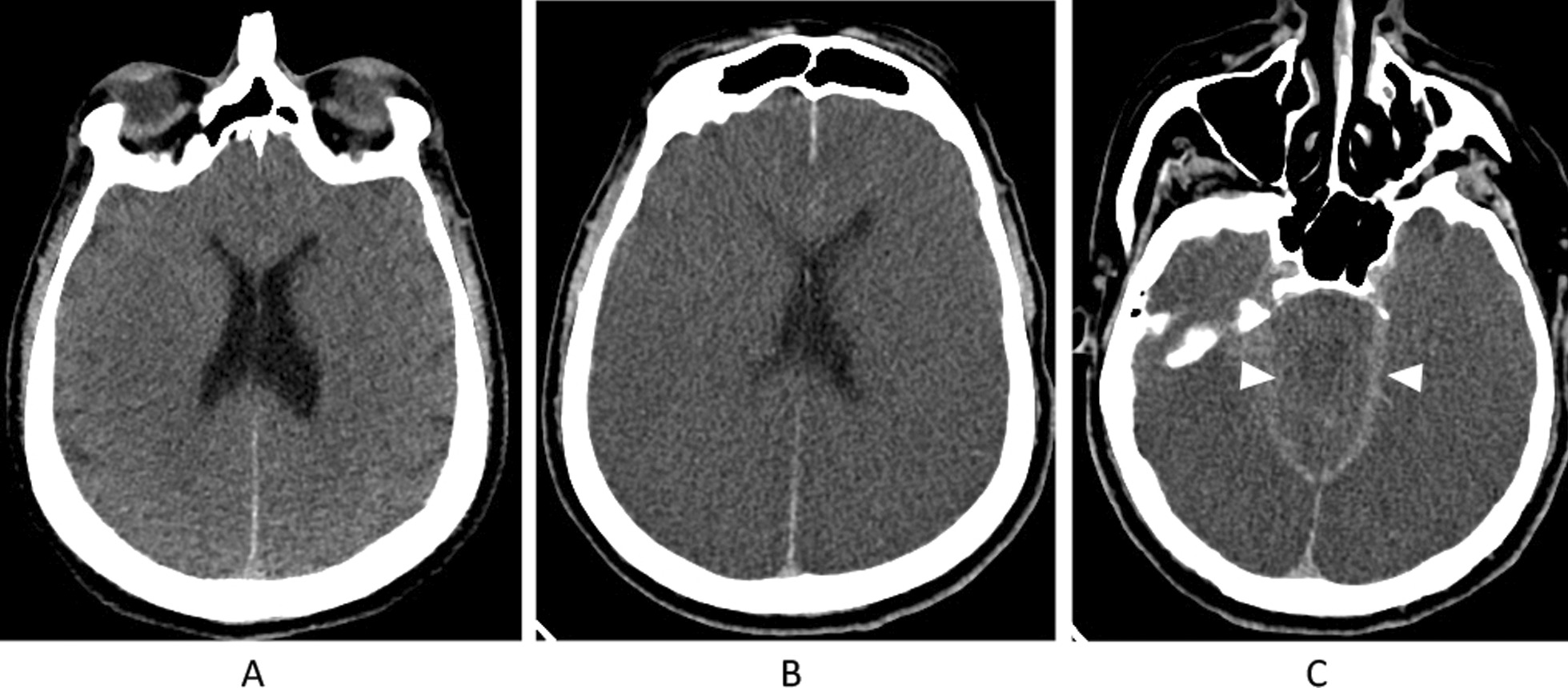
Imaging characteristics of catastrophic brain injury. Selected computed tomography (CT) images of a patient who presented to our hospital following cardiac arrest with anoxic brain injury. Initial non-contrast CT image obtained less than 2 h following initial arrest (**A**) demonstrates early loss of grey–white matter differentiation of the cerebral cortex. Follow-up study 36 h later (**B** and **C**) demonstrates progression of loss of grey white matter differentiation including the visualized brainstem with increased cerebral edema, sulcal and ventricular effacement and effacement of the basilar cisterns (arrowheads)

See Fig. 1 for an imaging example regarding the importance of neuroimaging that establishes a catastrophic brain injury before declaring BD/DNC. Briefly, this patient presented to the emergency department following cardiac arrest, likely due to an acute coronary ischemia syndrome. Advanced cardiac life support (ACLS) was performed for over 30 min before return of spontaneous circulation was persistently achieved. Initial neurological exam revealed a comatose patient with fixed, dilated pupils and loss of all brainstem reflexes. No sedating medications were given. Initial computed tomography (CT) imaging on arrival was consistent with diffuse anoxic brain injury but with preservation of cerebral and brainstem structures without herniation. Roughly 36 h following presentation, the patient made no clinical improvement, and another CT brain was requested. This time, it revealed substantial progression of diffuse cerebral edema with bilateral uncal herniation. At this time, neuroimaging was thought to satisfactorily explain the patient’s clinical state and BD/DNC was ultimately declared.

## Clinical exam in the determination of BD/DNC

The clinical determination of BD/DNC is detailed and can be daunting even to experienced critical care providers and neurologists. Correct diagnosis is of utmost importance, and the minimum clinical criteria and examination involves many steps. However, with proper training and preparation, including the use of checklists, success can be consistently achieved. For helpful checklists, see the 2010 American Academy of Neurology update on determining BD/DNC in adults [17], or the detailed checklist recently published by the World Brain Death Project, supplement 15 [6].

### Prerequisites and confounders

It is of paramount importance to ensure the etiology of brain injury, history, exam, and neuroimaging all are consistent with irreversible catastrophic injury to the whole brain. This involves exclusion of confounding variables that may cause the illusion of BD/DNC when this is in fact not the case. Potential confounders are vast and can be thought of by placing them into general categories such as clinical disease states (demyelinating polyneuropathy [18–20], botulism [21]), hemodynamics and body temperature [22], metabolic derangements [23], toxicities [24–29], sedation effects [30], and other medication effects). Extensive descriptions of potential confounders are beyond the scope of this review and have been described elsewhere [6, 31].

It should be noted that medications, even those that traditionally may not be thought of as sedating, can lead to comatose states or brainstem areflexia, particularly in the context of other systemic injuries. The half-life of each relevant medication should be known, and it is recommended to wait for sufficient clearance prior to proceeding with a clinical brain death evaluation. The minimum number of half-lives should be at least five [32]. It should also be noted that hepatic injury, renal injury, age, obesity, or hypothermia may delay clearance of substances by many hours or more. For some drugs, measurable blood levels are easily collected, but not for others. Unfortunately, blood pressure and temperature parameters to be met are not standardized worldwide, but a conservative recommendation for adults would require a systolic blood pressure > 100 mmHg and temperature > 36 °C before proceeding with clinical testing, consistent with the current American Academy of Neurology guidelines [6]. These criteria may be met by use of medications such as vasopressor support, or warming devices.

### Establishment of a persistent, irreversible cause

Once the above criteria have been established, it must be proven that the brain injury is irreversible, meaning that loss of function is complete and constant over time. An observation period should be utilized, for which standardized times are not well established across jurisdictions. However, in the setting of resuscitation following cardiac arrest, with or without TTM, at least a 24-h waiting period is recommended because there may be delayed recovery of brainstem function. In general, if there is any uncertainty regarding the irreversibility of the condition, further observation is recommended to exclude any doubt.

### Clinical testing: brainstem reflexes

After establishing a comatose state with complete unresponsiveness to maximal stimuli, determination of BD/DNC includes assessment for loss of brainstem reflexes, as follows: loss of pupillary responsiveness, loss of corneal, oculocephalic, oculovestibular, gag, and cough reflexes, absence of facial movement to noxious stimuli, and absence of cerebrally mediated movement to noxious stimulation of the extremities [6, 17, 33, 34]. Performance of each of these clinical tests requires attention to proper technique and experience. Clinical pearls regarding the performance of each test can be found below. A general recommendation is that the presence of a condition that would preclude performance of a brainstem reflex test, such as severe facial trauma or swelling, should necessitate ancillary testing [6]. The only exception for this would be the oculocephalic test (OCR), which may be omitted if there is a question of cervical spine integrity, such as in the setting of trauma or potential for ligamentous instability; however, the oculovestibular (OVR), or “cold caloric” test, must always be performed unless contraindicated.

#### Clinical pearls: pupillary light reflex

Traditionally, the pupillary light reflex can be obtained by use of a flashlight and the naked eye. However, the assistance of a magnifying glass or quantitative pupillometry is strongly recommended. Quantitative pupillometry provides quantitative, standardized information on the size and constriction speed of the pupil, is more reliable than subjective measurements [35], and has shown utility as a prognostic tool in comatose patients recovering from cardiac arrest [36] and in patients undergoing veno-arterial extracorporeal membrane oxygen therapy (VA-ECMO) [37], although it has not been validated for use in brain death and should not be used in isolation. Classically, pupils should be mid-sized and mid-position, although the exact pupil diameter consistent with BD/DNC is unknown and smaller pupils may be consistent as well, depending on the site of greatest neurologic injury. However, very small pupils (< 2 mm) should alert the practitioner to a possible confounder, e.g. from opiate intoxication or isolated brainstem injury [38].

#### Clinical pearls: corneal reflex

A definitive corneal reflex test should be performed by touching a cotton swab on a stick such as a Q-tip to the outer edge of the iris, applying enough pressure to depress the globe. Attempts to use lash stimulation or a drop of sterile saline may be useful as a screening tool, but are not definitive or sufficient in isolation to rule out the presence of a reflex. Care should be taken not to damage the cornea. In an absent reflex, no eyelid movement is seen.

#### Clinical pearls: oculocephalic reflex (OCR)

The head is moved horizontally to both sides. In an absent reflex, there is no movement of the eyes relative the head. OCR can also be tested vertically if desired. If a spinal cord injury or cervical spine instability has not been ruled out, this test should not be performed.

#### Clinical pearls: oculovestibular reflex (OVR)

After elevating the head to 30 degrees and ensuring a clear pathway to an intact tympanic membrane, instill ice cold water into the ear canal with a syringe attached to a catheter for 60 s. The absence of an OVR will reveal no movement of the eyes. In a comatose patient with an otherwise intact brainstem, the eyes will deviate toward the irrigated ear, with nystagmus beating in the opposite direction. After 5 min, allowing for re-equilibration of the temperature of the endolymph on the tested year, test the contralateral ear. OVR testing should be avoided if there is severe basal skull trauma, as it may compromise the reflex response, or may physically disrupt the ear canal or tympanic membrane. Presence of severe orbital trauma may affect free range of motion of the globes, and can preclude successful OVR or corneal reflex testing, necessitating ancillary testing.

#### Clinical pearls: gag and cough reflex

Using a suction catheter or tongue depressor, stimulate the posterior pharyngeal wall bilaterally. To test a cough reflex, stimulate the trachea near the carina with use of a deep endotracheal suction catheter, typically found connected to the endotracheal tube apparatus. The absence of a reaction to both tests is consistent with BD/DNC. Of note, the phrenic nerve is responsible for parts of the efferent limb of the cough reflex, thus if there is concern for a high cervical injury, this could obliterate this reflex and an ancillary test should be performed.

#### Clinical pearls: motor testing

Apply deep pressure to the following points: the condyles at the level of the temporomandibular joints, the supraorbital notches, the sternal notch, and all four extremities proximally and distally. These measures should not elicit any movement that is not considered to be spinally mediated. Differentiation of spinal- from brain-mediated movements is often challenging, and requires an experienced provider. If results remain unclear, an ancillary test is required. In-depth review of all spinally mediated movements is beyond the scope of this review but can be found here [6]. High-yield examples include triple flexion, decerebrate-like (extension) movements, Babinski sign and fasciculations [39, 40]. The presence of severe neuromuscular disease or facial trauma necessitates an ancillary test, as these conditions potentially mask motor responses.

#### Clinical testing: apnea

The goal of apnea testing is to create a buildup of carbon dioxide that maximally stimulates the medullary respiratory centers, which are ultimately triggered by the ensuing acidic pH of the cerebrospinal fluid (CSF). Prior to testing, perquisites must be met, including ensuring absence of clear spontaneous respirations, normotension (systolic blood pressure ≥ 100 mmHg or mean arterial pressure ≥ 60 in adults), normothermia (temperature ≥ 36 °C), absence of hypoxia, and eucapnia [6, 17, 33, 34]. In the severely brain injured patient, meeting prerequisites can be challenging, and one study found these conditions preclude apnea testing in up to 7% of patients [41].

Prior to performing the apnea test, ensure that the patient is not breathing over the set ventilator rate. Providers should be aware that the ventilator may be auto-triggered by non-respiratory movements or ventilator factors such as condensation in the tubing, or endotracheal tube leaks [42–45]. Readily available medications, including intravenous fluids, vasopressors, and warming devices should be considered based the active medical issues of each individual patient. Use of an arterial line is also strongly encouraged to ensure arterial blood gas and continuous blood pressure measurements are easily obtained.

The typical procedure involves disconnection from the ventilator while monitoring for signs of spontaneous respirations [6, 17, 33, 34]. The patient’s chest and abdomen should be exposed to assess for any respiratory effort during testing. Prior to testing, ventilator settings should be adjusted such that PaCO_2_ ranges between 35 and 45 mmHg or 4.7–6.0 kPa (eucapnia or mild hypercapnia). The patient is then pre-oxygenated with 100% oxygen for 10–15 min to a goal PaO_2_ of > 200 mmHg. Adequate oxygenation is ensured during apnea testing by inserting a catheter through the endotracheal tube to the level of the carina, delivering oxygen at 4–6 L/min, which is continued throughout the duration of testing. Care should be taken to ensure that the diameter of the catheter is < 70% of the internal lumen of the endotracheal tube, so that barotrauma is not inadvertently caused. If there are no spontaneous breaths, an ABG is measured after eight to ten minutes, and if PaCO_2_ rises to ≥ 60 mmHg, the apnea test is considered positive. Caution should be taken in patients who chronically retain CO_2_ such as chronic obstructive lung disease, and CO_2_ targets may need to be adjusted depending on the baseline level; in this setting, the target PaCO_2_ should be at least 20 mmHg above the known elevated baseline value (as well as ≥ 60 mmHg). If the CO_2_ target is not reached, the test can either be continued for another 5 min, or can be repeated for a longer period of time. If the apnea test cannot be attempted at all due to cardiac or pulmonary instability, or is aborted due to instability during testing, an ancillary test should be performed.

Because apnea testing carries some inherent risk of hemodynamic or pulmonary compromise, it is generally performed as the last clinical test. Potential complications include hypotension, hypoxemia, arrhythmia, barotrauma, or cardiac arrest [41, 46–51]. Because of this, experienced practitioners as well as appropriate medications should be at the bedside to monitor and treat potential complications. Estimations of aborted apnea tests due to complications range from 1.6 to 4.8% of cases [46, 47, 52].

### Number of examinations

The number of exams, examiners and the time interval between exams is variable among countries and jurisdictions, but typical BD/DNC testing involves between 1 and 3 exams [33, 53, 54]. In the United States, only one exam is currently required in adults [17]. In some countries, BD/DNC must be performed by two physicians, while in others, two separate exams by two different physicians are required [55]. If two exams are performed, we recommend against a waiting period, and recommend performance of only one apnea test in adults [6].

## Ancillary testing

### Indications

The mainstay of diagnosis of BD/DNC rests on the above described criteria, that is: establishment of a clear, irreversible cause of brain injury, exclusion of confounders, persistent coma, clinical assessment of brainstem reflexes, and apnea testing. If a patient can be determined BD/DNC based on clinical criteria, ancillary testing is not needed. That said, a myriad of circumstances can ultimately lead the provider to cast doubt on the diagnosis because of factors such as inability to complete a clinical test, inability to exclude confounders, or lack of clarity in the interpretation of a particular test. In these circumstances, ancillary testing is recommended [6]. In fact, when the Harvard Brain Death Criteria were initially proposed, EEG was recommended for every evaluation [2]. To this day some countries still require use of an ancillary test [11]. While this is not recommended by the majority of countries and professional societies, ancillary testing remains a commonly utilized and relied-upon tool in the BD/DNC evaluation. Even in cases where it is clear that an ancillary test will be required, the clinical examination should still be completed to the fullest extent possible, and any signs of life during this testing would preclude ancillary testing, as the patient would clearly not be BD/DNC.

In general, a useful ancillary test can be thought of as having the characteristics of an ideal biomarker: it should be noninvasive, easily measured, inexpensive, produce rapid results, have high sensitivity and specificity, and should aid in prognostication. In particular, ancillary tests should not have false positives that could lead to the inappropriate diagnosis of BD/DNC, and should not be subject to confounders such as sedation effects [56]. However, no ancillary tests to date satisfy all these criteria, and the risks and benefits of each must be considered on a case-by-case basis depending on the unique circumstances of each clinical case. Below we review the most commonly utilized ancillary tests and make general recommendations regarding the use of each. See Table 1 for a brief description of recommended ancillary tests, all based on cerebral blood flow.

**Table 1:** Recommended ancillary testing

Test	Procedure	Comments
Digital subtraction angiography (DSA)	Lack of arterial contrast opacification where the carotid and vertebral arteries enter the skull correlates to absence of perfusion External carotid circulation will appear intact	Historically considered the gold/reference standard. Limited by available expertise, skill, cost, and transfer to an operating room/angio suite Limited by decompressive procedures that may lower intracranial pressure
Radionuclide imaging	Lipophobic or lipophilic technetium-based compounds produce signal as they circulate intracranially (lipophobic), or pass through the blood–brain barrier and are metabolized by metabolically active parenchyma (lipophilic)	Lipophobic compounds inadequately demonstrate flow through the posterior fossa, thus lipophilic preferred Tomographic processing of lipophilic compounds is commonly known as single photon emission computed tomography (SPECT) and is increasingly used as a reference standard, but cannot be done at the bedside
Transcranial Doppler (TCD)	Allows measurement of dynamic changes to brain blood flow and confirms circulatory arrest when performed in the anterior and posterior circulations Systolic spokes and oscillating flow appearance indicate obstruction to blood flow	Portable, easily performed at the bedside 10% of patients have inadequate bone windows, thus the absence of a waveform necessitates reference to a previous study that demonstrated perfusion 2 exams suggested at least 30 min apart Limited by decompressive procedures that may lower intracranial pressure Not suggested in pediatric patients

### Digital subtraction angiography (DSA)

DSA is considered the gold standard in ancillary testing, with reports of 100% sensitivity and specificity [57, 58]. Lack of contrast opacification during 4-vessel cerebral angiography at the level that the vessels enter the skull base, with intact extracranial circulation, indicates lack of perfusion to the brain and establishes BD/DNC in the setting of an otherwise consistent clinical exam. Similar to other ancillary tests of blood flow, including CT angiography (CTA), magnetic resonance angiography (MRA), and transcranial Doppler (TCD), flow dynamics are impacted when there are procedures that decompress the brain such as EVD or craniectomy and can complicate interpretation [59]. Further limitations and complications of DSA include time, transfer to the angiography suite, the need for technical skill, risk of vasospasm, and contrast nephropathy [60, 61].

### Single photon emission computed tomography (SPECT)

As a radionuclide study, SPECT involves introducing a radiotracer (usually technetium 99 compounds) into the peripheral circulation, and, in the case of lipophilic Technetium-based compounds, diffusion across the blood–brain barrier, uptake into the brain, and metabolic breakdown [62]. Similar hydrophobic compounds can also be used but are not preferred given they remain intravascular and have no bearing on metabolic activity of the brain parenchyma [63, 64]. Images are then converted by tomographic processing into a SPECT image. Although this modality typically requires transfer to a radiology suite or nuclear medicine department, it is still thought to be less resource- and time-intensive than DSA [65], with similarly high sensitivity and specificity [66, 67]. SPECT has grown in popularity, and is considered a reference standard similar to DSA [64, 67, 68] One major limitation includes potentially inadequate visualization of the posterior fossa which could lead to false-positive results, a phenomenon that improves with tomographic processing [69, 70].

### Transcranial Doppler (TCD)

TCD presents an attractive tool for ancillary testing given its overall ease of use at the bedside, low expense, and potential for visualization of the posterior circulation [71]. Typically, 2 separate exams, both anterior and posterior, separated by at least 30 min are required. It involves the use of acoustic temporal bone windows for analysis of the anterior circulation, but also requires evaluation of the posterior circulation as well when used for evaluation of BD/DNC. Patterns of flow detected by TCD are seen as systolic spikes with reversal of flow in diastole which suggests infraclinoid carotid obstruction or posterior circulation obstruction, and biphasic or oscillating flow velocities that indicate terminal carotid obstruction [71, 72]. Limitations on use of TCD include reliance on bone windows, given TCD is naturally limited in roughly 10% of the population that have inadequate temporal windows [73]. The absence of waveforms is not sufficient to make the diagnosis. Use of other windows such as transorbital and transcervical can be employed in this case but are not as well established [73, 74]. Interpretation is also dependent on technical expertise, although likely less so than DSA or SPECT.

### Computed tomographic angiography (CTA)

Given its ease of use, speed, and wide availability, there has been much enthusiasm for the use of CTA in ancillary testing, but data is limited and its use as an ancillary test is not currently recommended. Although CTA has been recommended by some countries, it is not recommended as an ancillary study in the U.S. at this time [6, 17]. The general concept involves peripheral (venous, as opposed to DSA which utilizes arterial) injection of iodinated contrast and evaluation of blood flow to the distal cerebral vasculature after a specific time period (usually 25–40 s), including the A3 division of the anterior cerebral artery (ACA), the M4 division of the middle cerebral artery (MCA), P2 division of the posterior cerebral artery (PCA), basilar artery, and sometimes the internal cerebral veins and great cerebral vein (Galen) depending on the specific methodology utilized [75, 76]. Absence of flow in the intracranial circulation with persistence in the extracranial carotid circulation is consistent with BD/DNC.

A primary problem in the interpretation of CTA is that of so-called “stasis filling”, which is contrast opacification of a vessel that is initially impeded by intracranial pressure, then nevertheless is observed in the distal vasculature in the absence of actual perfusion [76, 77]. Stasis filling is not fully understood but thought to be related to contrast timing and intracranial vessel length. Further questions arise regarding optimal contrast timing and measurements of meaningful perfusion [78]. Image interpretation often proves difficult, as it can be difficult to determine the specific cerebral vessels most likely to produce an accurate, reliable result. To this end, there are still no agreed-upon technical protocols for use of CTA, and further studies and consensus are needed before it can be recommended as an ancillary test [79–81].

### Magnetic resonance imaging (MRA) and angiography (MRA)

MRI/MRA, similar to CTA, shows promise in ancillary testing; however it is subject to similar pitfalls as other flow-based studies, such as CTA, and is also not recommended for use in diagnosis of BD/DNC. Unlike CT, MRI shows greater resolution and detail regarding the extent of neurologic damage, and sheds more light on possible causes that may have previously been unclear, although it plays no particular additional role as an ancillary test. Similar to CTA, MRA is subject to pitfalls of stasis filling [82], requires evidence of flow in the external carotid artery to diagnose BD/DNC [83, 84], and may be difficult to interpret in the setting of procedures that reduce craniovascular pressure, such as craniotomy [82]. Compared to CTA, MRA although widely available, has the disadvantage of increased time, expense, contraindications due to metallic implants, and concerns regarding nephrogenic systemic fibrosis [82].

### Electroencephalography (EEG) and evoked potentials (EP)

As opposed to the studies above that rely on visualization of blood flow, EEG has the ability to detect electrical activity, and as one of the first neurologic tests in general, it has long been used to augment the clinical determination of BD/DNC [85, 86]. However, EEG has perhaps proved more valuable in cases that aim to detect subtle meaningful residual cerebral activity, such as covert consciousness, rather than to exclude the presence of meaningful cerebral function [16, 18, 19, 87]. As such, EEG is not recommended as an ancillary test in adults unless otherwise required by local laws or protocols. In the evaluation of BD/DNC, EEG is limited by its ability to detect only cortical activity reliably [86, 88], and lack of ability to assess the posterior fossa/brainstem. In general, and particularly in an ICU setting, interpretation is limited by a number of artifacts, leading to potential false negatives, and EEG activity may be artificially suppressed in a number of clinical scenarios including TTM or sedation, leading to false positives [86, 88, 89].

Recent advancements in invasive neuromonitoring, including use of continuous electrocorticography with subdural or intraparenchymal electrodes shed light on the value of EEG not in ancillary testing, but rather as an aid to understanding the electrophysiological markers that ultimately lead to brain death. Electrocerebral silence on traditional scalp EEG is a crude marker of BD/DNC and further neurophysiological markers are needed to elucidate the precise timing of the toxic cascade of events that leads to irreversible global brain injury. Terminal spreading depolarization, or a depolarization wave that marks the commitment point during which neurons initiate a toxic physiological cascade leading to death, has been widely studied in animals. This was recently demonstrated for the first time in an observational study of nine patients with catastrophic brain injury, through placement of subdural and intraparenchymal electrodes [90]. Similar to animal models, this study demonstrated a cascade of events following circulatory arrest, starting with a decline in brain tissue partial pressure of oxygen, followed by nonspreading depression, likely related to a cerebral oxygen sensory that shuts down neuronal activity in response to low partial pressure of oxygen. After tens of seconds this was ultimately followed by terminal spreading depolarization [90]. Further elucidation of this fundamental end-of-life process could have value in tailoring resuscitation efforts, and neuroprognostication.

EP includes visual evoked potentials (VEP), somatosensory evoked potentials (SSEP), and/or and auditory evoked potentials (AEP). EPs in general have been proposed as a complement to EEG, given their ability to evaluate the integrity of an entire pathway, from peripheral stimulus to cortical output, particularly of the brainstem [91–93]. Although they also rely on interpretation by a skilled provider, they require less time and resource input. In particular, EEG and EP studies may be helpful in the setting of intracranial decompressive procedures which, as previously discussed, can confound interpretation of flow-based imaging studies [94]. Of note, each EP study only studies the specific pathway of the test (e.g., visual, sensory or auditory), and thus is not assessing the integrity of other pathways.

## Special considerations: BD/DNC and extracorporeal membrane oxygenation (ECMO)

Over the last 2 decades, the use of ECMO has rapidly expanded thanks to landmark trials that support its mortality benefit [95–97]. With increased utilization, it naturally follows that the number of BD/DNC evaluations in ECMO-supported patients has also increased in recent years, both due to the inherent complications of ECMO circuits as well as the underlying disease processes themselves [98]. Furthermore, given that the use of veno-arterial ECMO (V-A ECMO) bypasses the pulmonary and cardiac circuits, its use effectively prevents arrest of cardiopulmonary function, and necessitates BD/DNC as the primary determination of death. In fact, one study published in 2009 found that of all patients treated with ECMO, 21% were eventually declared BD/DNC [99].

In general, the BD/DNC evaluation is performed similarly in an ECMO patient. Apnea testing is still recommended, but unique technical aspects should be considered regardless of use of veno-venous (V-V) or V-A methods. Providers should continue to establish a cause of the neurological state, complete prerequisites, and proceed through the same clinical testing as any critically ill patient. Interestingly, in a recent study of those declared brain dead on ECMO, 42% did not undergo an apnea test, although this can still be performed safely in most ECMO patients [100].

The basic concept of apnea testing remains the same: pre-oxygenation, observation for spontaneous breaths, and measurements proving the buildup of CO_2_ in arterial circulation. Pre-oxygenation can be performed in a similar fashion to non-ECMO patients in that a catheter delivers 100% FiO_2_ to the level of the carina, with the option of utilizing CPAP or PEEP to maintain recruitment, while adjusting the ECMO gas flow to 100% FiO_2_. The added steps involve minimizing the sweep gas flow rate (CO_2_ clearance rate) to 0–1 L/min in order to prevent the exchange of CO_2_ for oxygen in the membrane oxygenator. All other factors being equal, this allows buildup of CO_2_ in the arterial circulation [101–104].

It should also be noted that in V-A ECMO, due to the phenomenon of “mixing” occurring when residual native circulation allows antegrade flow from through the left ventricle into the aorta and mixes with retrograde flow from the arterial cannula, distal arterial measurements may be inconsistent with those from the membrane oxygenator circuit, and should be collected simultaneously to avoid inconsistencies [105]. The targets for pH and CO_2_ levels should be the same for both sites, and are recommended to be pH < 7.3, and PaCO_2_ ≥ 60 mmHg. The indications for ancillary testing in ECMO patients, and interpretation of different types of testing, are not well studied, and particular caution should be taken with use of TCD, as it relies on measurement of pulsatile flow [106].

## Special considerations: targeted temperature management (TTM)

Use of TTM in patients who suffer cardiac arrest, particularly out of hospital cardiac arrest (OHCA), has been studied for years. Theoretical benefits include basal cerebral metabolism reduction, prevention of free radical formation, reduction of reperfusion injury, and suppression of neuronal death pathways. Current guidelines recommend use of TTM in patients who remain comatose following (OHCA), but the optimal temperature remains unclear [107]. Previously, it was thought that at least moderate hypothermia (≤ 35 °C) offered greater neuroprotection and improved neurologic outcomes compared to standard treatment with normothermia based on two seminal trials published in 2002 [108, 109]. Eleven years later, the first TTM trial showed no difference in mortality with a target temperature of 33 °C versus 36 °C [110]. This led to a relaxation of temperature targets internationally, and in some cases, abolishment of TTM protocols altogether [111, 112]. Publication of the recent TTM2 trial similarly showed no significant difference in survival between 33 °C and normothermia (≤ 37.5 °C) [113]. Additionally, a recent meta-analysis that included 10 randomized clinical trials studying TTM in OHCA similarly found that targeting deep, moderate, or mild hypothermia may not improve survival or outcomes in OHCA [114]. It should be noted that in both TTM trials, average survival rates of OHCA proved to be well above historical averages regardless of treatment group, and there were similar rates of pharmacologic intervention and cooling device usage among both groups, supporting the notion that close regulation of temperature and avoidance of fever is critical, regardless of the target temperature [110, 113]. There also continue be questions regarding whether certain patients more likely to have a poor outcome, such as those with prolonged or unknown down times or non-shockable rhythms, might benefit from at least mild hypothermia given the heterogeneity of patient selection in the various TTM trials [114, 115].

Although there are persistent debates surrounding the general use of TTM and target temperature in OHCA, TTM use has expanded to other neurologic conditions including cerebrovascular disease and traumatic brain injury (TBI) [116]. Additionally, there will continue to be clinical scenarios where mild or moderate hypothermia is reasonable, and neurologists and critical care providers should be aware of the effects of TTM on the determination of BD/DNC.

Some of the most important physiologic effects of therapeutic hypothermia include blunting of brainstem reflexes [117, 118], decreased clearance of medications (particularly if there is concomitant hepatic or renal injury) [17, 119, 120], and false-positive electrocerebral silence on EEG [22, 121]. The exact extent of the effect of these confounders is unclear for the individual patient, given variation in target temperature, medication use and variable evidence of end-organ injury.

As such, a standardized approach to the TTM patient is suggested. Reports of patients who have seemingly recovered some neurologic function after being incorrectly declared brain dead following TTM reveal that providers have generally not closely followed consensus protocols regardless of the use of TTM and BD/DNC evaluation [120]. In terms of a waiting period, it is suggested that evaluation for BD/DNC not be initiated until at least 24 h following complete rewarming to allow for normalization of existing brainstem reflexes [6, 17]. Similarly, if sedating medications are used, it is recommended to wait at least 5 half-lives until the start of clinical testing (longer if hepatic or renal insufficiency), and to collect serum levels of sedating medications if available to ensure they are at least below therapeutic [6, 17]. Additionally, particularly if there are questions regarding the former two points, an ancillary blood flow study can be performed.

## Special considerations: pediatric BC/DNC

When considering pediatric populations, many of the same principles in the determination of BD/DNC apply. However, unique aspects of pediatric anatomy and physiology, as well as a general paucity of high quality studies in this sub-population lead to unique considerations and an overall additionally cautious approach. See Table 2 for a detailed comparison of adult versus pediatric brain death protocols based on current guidelines in the U.S. [4, 17].

**Table 2:** Differences in BD/DNC guidelines between adults and children

	Infants and children	Adults
Definition	States Uniform Determination of Death Act definition Uses term “brain death” Definition of brain death provided	States Uniform Determination of Death Act definition Uses term “brain death” Definition of brain death provided
Evidence-based	Yes Patients who recover function addressed	Yes Patients who recover function addressed
Qualifications	States examiner must be attending physician competent/qualified to perform brain death evaluation Specifies a standardized checklist should be used	States that all physicians making a determination of brain death be intimately familiar with brain death criteria and have demonstrated competence in this complex examination Specifies a standardized checklist should be used
Prerequisites	Establish cause of coma Establish that brain injury is irreversible Therapeutic hypothermia discussed—no specific waiting period given Consider deferring BD evaluation for 24–48 h after resuscitation When in doubt, observe and postpone BD evaluation Exclude mimicking conditions Physiologic parameters normal for age Metabolic derangements need correction Neuromuscular blockade addressed (recommends train of four testing if recently given) Drug intoxication (tables provided for elimination ½ life, says may need to wait several ½ lives) Temp > 35 °C	Establish cause of coma Establish that brain injury is irreversible Therapeutic hypothermia discussed—no specific waiting period given Ensure certain period of time has passed to exclude the possibility of recovery (usually several hours) Exclude mimicking conditions Physiologic parameters normal (SBP ≥ 100) Metabolic derangements need correction Neuromuscular blockade addressed (recommends train of four testing if recently given) Drug intoxication (wait 5 ½ lives) Temp > 36 °C
Neurologic examination	Number of examinations: 2 (The first examination determines the child has met neurologic examination criteria for brain death. The second examination, confirms that the child has fulfilled criteria for brain death.) Observation period 12 h (if age > 30 days) Observation period 24 h (if age 37 weeks estimated gestational age to 30 days) 2 different attending evaluators Complete neurologic exam: no mention of oculocephalic reflexes, mentions primitive reflexes for neonates/infants Discusses spinal reflexes	Number of examination: 1 Observation period: none Complete neurologic exam: OCR and OVR listed, mentions c-spine injury, details on OVR procedure provided Discusses spinal reflexes
Apnea testing	Mentions prerequisites 2 apnea tests required Both tests can be done by same attending Specifies 5–10 min of pre-oxygenation Specifies high c-spine injury as contraindication Recommends T-piece or self-inflating bag. Discusses problems with tracheal insufflation using catheter in ETT and problems using CPAP on ventilator Criteria: no respiratory effort, PaCO_2_ ≥ 60 and ≥ 20 rise from baseline Stop apnea test: SaO_2_ < 85% or hemodynamic instability (no specifics)	Mentions prerequisites, includes no prior evidence of CO_2_ retention 1 apnea test Specifies 10 min of pre-oxygenation Specifies starting PaO_2_ ≥ 200, drop rate to 10, PEEP to 5 Recommends tracheal insufflation using catheter in ETT Specifies length of 8–10 min of apnea Criteria: no respiratory effort, PaCO_2_ ≥ 60 or ≥ 20 rise from baseline Stop apnea test: SaO_2_ < 85% for 30 s, retry with CPAP 10 or SBP < 90
Ancillary testing	Acceptable reasons to use ancillary testing When components of the examination or apnea testing cannot be completed safely due to the underlying medical condition of the patient If there is uncertainty about the results of the neurologic examination If a medication effect may be present To reduce the inter-examination observation period Ancillary studies may also be helpful for social reasons allowing family members to better comprehend the diagnosis of brain death Acceptable tests: angiography, EEG, radionucleotide CBF Pharmacologic agents that could affect the results of testing should be discontinued and levels determined as clinically indicated. Low to mid therapeutic levels of barbiturates should not preclude the use of EEG testing Tables detailing EEG and CBF diagnostic yields in brain death States if ancillary study not consistent with brain death, do not necessarily need to repeat with subsequent evaluation	Acceptable reasons to use ancillary testing When uncertainty exists about the reliability of parts of the neurologic examination When the apnea test cannot be performed Acceptable tests: angiography, EEG, nuclear scan Discussion of diagnostic yield of various tests in brain death
Death declaration	Time of death not specified Discusses addressing BD with families, effective communication, supporting families through the process, requests for ongoing organ support	Time of death: time of blood gas with appropriately elevated CO2 or time ancillary test results

The minimum age to determine BD/DNC varies by country, ranging from 36 to 37 weeks gestation [5, 122]. Regardless of age, far fewer patients are declared dead in this population, estimated as 1:100 compared to adults [123].

It is important to first understand physiologic differences in pediatric populations to understand differences in the determination of BD/DNC. First, formation of the pediatric skull necessitates the presence of patent sutures and open fontanelles, allowing displacement of brain parenchyma and altering CSF dynamics, thus complicating a number of aspects of BD/DNC evaluation including response of the parenchyma to elevated intracranial pressure, and the interpretation of blood flow studies conducted as ancillary tests, similar to evaluation of an adult patient that underwent a decompressive procedure [5]. Secondly, tracheal insufflation has the potential to cause barotrauma given the small and delicate airways in pediatric patients, and is not recommended in newborns [5]. The presence of inborn errors of metabolism should be considered when newborns present with coma, as well as congenital or secondary causes of renal or hepatic dysfunction that may delay clearance of sedating medications [6].

Unlike in the adult population where TTM is used almost exclusively in cardiac arrest patients with temperature targets usually considered mild hypothermia (36 °C), (particularly following the first TTM trial), in pediatric protocols, at least moderate hypothermia is used in the setting of neonatal asphyxia, often ≤ 35 °C [124]. Providers should be aware of the effects of hypothermia on delayed clearance of sedative medications as well as blunting of brainstem reflexes, as described above.

Given the general lack of high quality data to guide management of pediatric patients, a conservative approach is taken and 2 exams are recommended, often including 2 apnea tests. In some cases, a 24-h waiting period is recommended between exams, although arguably the most important waiting period is prior to any clinical determination if there is any concern for reversibility of the condition [5, 122].

Ancillary testing is treated similarly to adult patients, and is generally pursued when a complete exam is unable to be performed or when apnea testing is not able to be attempted. Similar concepts to those described in prior sections apply. In general, DSA is rarely pursued given the lack of technical expertise at most centers, and nuclear perfusion imaging (SPECT) is the most preferred. As mentioned above, interpretation of other cerebral blood flow studies such as CTA, MRA or TCD becomes more complicated in the infant population, and are not yet validated for use in BD/DNC evaluation [5, 122].

## Controversies in BD/DNC determination

Decades following the first conceptions of brain death, BD/DNC is now widely accepted philosophically, religiously, and medicolegally throughout much of the world. However, many dilemmas in the field persist, which, unlike many fields of medicine, span religious, cultural, and scientific realms.

Perhaps the greatest source of controversy exists around the worldwide variability in the determination of BD/DNC. In the largest review of world protocols to date, Lewis et al. successfully communicated with practitioners from 69% of all world countries, and found that of these, 61% had protocols for the determination of BD/DNC (42% of the world) [11]. As discussed above, most protocols reviewed adhered to the concept of whole brain death (87%), and a minority referred to brainstem death (14%). Furthermore, countries differed in many areas of determination of BD/DNC including prerequisites, imaging, components of the clinical exam, apnea testing and ancillary testing, among other differences [11]. These findings are consistent with multiple prior studies based on survey data of practitioners across the world [33, 53, 54, 125]. Even within nations such as the U.S. where the American Academy of Neurology (AAN) practice parameters in the determination of brain death are meant to service as a clear guide to institutions, there was significant variability found in many of the major categories of determination [53]. Lastly, training in evaluation of BD/DNC is often lacking, evidenced by surveys that demonstrated lack of understanding of the rationale and diagnostic testing for determination of BD/DNC [126], and performance of the exam [127]. It is imperative that determination of BD/DNC be standardized as much as possible throughout the world, including among institutions and among providers themselves in order to maintain public and professional confidence in brain death evaluations and ensure consistency. It should not be assumed that the presence of a national or institutional protocol is a surrogate for the details of a provider’s clinical examination and reasoning, and efforts should be made to improve all angles of the evaluation, including at the bedside level. Because of local laws, cultural values, and religious beliefs it is probably unrealistic to assume that BD/DNC be determined exactly in the same way across the world. Rather, a set of minimum standards based on review of the existing literature and expert consensus should be adhered to. This was the outcome of the recent World Brain Death Project, a set of international professional societies and leading researchers who formulated a set of consensus recommendations (see supplement on minimum clinical criteria) [6].

In conclusion, the concept of brain death has grown, been refined, and increasingly accepted by the scientific community and the public since its original conception in the 1950s. As the practice of critical care becomes ever more sophisticated with development of advanced life support measures, brain death evaluations will continue to be a major part of the practice of critical care medicine, and there will continue to be no shortage of ethical, technical, and medicolegal questions that will need to be navigated. There will continue to be a need for compassionate and competent critical care doctors who not only understand the intricacies of the brain death evaluation, but can communicate its results and subtleties to the lay public. Although considerations are vast when declaring a patient dead by neurological criteria, comfortability and confidence can be achieved. Because of the technical and high stakes nature of the brain death evaluation, education of trainees both in neurology and in general critical care is of utmost importance. We recommend prioritization of this training, mediated by experienced neurointensivists in residency programs, and advocate for increased use of direct observation at the bedside, simulations, development of other educational curricula, and international collaboration to address knowledge gaps.

Although controversies and questions remain in the field, we are encouraged by the continued robust discussion internationally regarding best practices in the declaration of BD/DNC. It is vital we continue to address inconsistencies in both philosophy and practice around the world to ensure as much as reasonably possible that the declaration of BD/DNC is fair, consistent, and equitable around the world. Although there may always be some level of variation given international differences in culture and religious values, we advocate for a minimum standard of criteria to serve as a foundation and a guide to clinicians everywhere.

## Data Availability

Data sharing is not applicable to this article as no datasets were generated or analyzed during the current study.
